# *In vitro* cellular interaction of drug-loaded liposomes with 2D and 3D cell culture of U87-MG cell line

**DOI:** 10.1371/journal.pone.0320374

**Published:** 2025-03-25

**Authors:** Tasneem Alsheleh, Manar Zraikat, Fadwa Daoud, Dana A. Alqudah, Sharif Abdelghany, Ahmed Abu Siniyeh, Walhan Alshaer

**Affiliations:** 1 Department of Biology, Faculty of Science, The University of Jordan, Amman, Jordan; 2 Department of Pharmacology, Faculty of Medicine, The University of Jordan, Amman, Jordan; 3 Cell Therapy Center, The University of Jordan, Amman, Jordan; 4 Department of Pharmaceutics and Pharmaceutical Technology, Faculty of Pharmacy, The University of Jordan, Amman, Jordan; 5 Department of Clinical Laboratory Sciences, Faculty of Science, The University of Jordan, Amman, Jordan; Queen’s University Belfast, UNITED KINGDOM OF GREAT BRITAIN AND NORTHERN IRELAND

## Abstract

The distinctive physiological and physical properties of 3D cultures that mimic tumor microenvironments *in vivo* make them more suitable for assessing the efficacy of drugs and nanoparticles compared to 2D culture models. Therefore, this study aims to examine and contrast how liposomes interact with cell cultures in both 2D and 3D models. Hanging drop technique was used to generate 3D spheroids. Cellular toxicity of Doxorubicin and Doxil^®^-liposomes was tested using an MTT assay. Cellular uptake of Doxil^®^-liposomes was investigated in 3D and 2D cell culture models using flow cytometry and confocal microscopy. Finally, migration and invasion assays were used to investigate the Doxil^®^-liposomes interaction with the two models 2D model and 3D model, respectively. Our findings show that cells were able to form spheroid structures when a specific cell ratio was maintained. Flow cytometry analysis revealed that 2D cells exhibited higher Doxil^®^-liposome uptake than 3D cells. The data obtained from confocal and fluorescent microscopy supported the findings of the flow cytometry analysis. Furthermore, the MTT assay showed that Doxil^®^-liposomes induced less metabolic-disruption compared to free Doxorubicin. Our results also demonstrated that Doxil^®^-liposomes interacted more loosely with the 3D model than 2D cells, which was further confirmed by measurements of the total migration and invasion areas. Therefore, a 3D model replicating the *in vivo* conditions of tumor structure and extracellular matrix to assess the delivery of liposomal-nanoparticles to spheroids through a collagen matrix can be more informative and recapitulate the *in vivo* microenvironment than the 2D model.

## 1. Introduction

Cancer is characterized by uncontrolled growth and proliferation, leading to the formation of malignant tumors that can invade adjacent tissues. Conventional chemotherapy lacks specificity, causing severe damage to both tumor and normal cells. This necessitates the development of more effective delivery systems to reduce the side effects and increase the efficiency of the antitumor drugs [1]. The use of nanotechnology in medicine, particularly for drug delivery, has rapidly expanded in recent years [2]. Nanoparticles have been used to improve the delivery of poorly water-soluble drugs by enabling faster dissolution in the bloodstream [3].

Cell culture systems have significantly impacted biomedical research by reducing reliance on animal models, leading to important discoveries in drug development and exploring different disease mechanisms. Historically, two-dimensional (2D) cell cultures have been widely used for several decades and are considered a cornerstone of *in vitro* research [4]. However, growing cells as monolayers on plastic surfaces fail to accurately replicate the *in vivo* conditions, especially the biological activity of therapeutic agents and cellular heterogeneity in oncological studies. This obstacle is related to the inability of the 2D cultures to replicate the three-dimensional (3D) microenvironments of tumor tissues, including biomedical signaling, structural organization, and cell-cell and cell-matrix interactions [5]. Researchers overcome these limitations by developing three-dimensional culture (3D) cell cultures, commonly referred to as 3D models, which exhibit greater physiological relevance and have led to advancements in our understanding of various biological mechanisms in healthy and diseased tissues [6]. These 3D cell culture systems can recapitulate key features of solid tumors that mimic the *in vivo* environment, including tumor morphology, gradients of chemical and biological factors, and dynamic and reciprocal interactions between tumors and their stroma. Consequently, offering insights into understanding cell behavior, drug penetration, and therapeutic responses.

Nanotechnology and its applications, like liposomal-based nanoparticles, have provided more effective systems for dealing with the native structure of the tumor. Nanoparticles can interact with the tumor mass and its surrounding vasculature via the enhanced permeability and retention effect (EPR). Their biocompatible and biodegradable structures can reduce toxicity and improve selectivity in therapeutic applications [7,8]. Doxorubicin (Dox), an anthracycline antibiotic, was first isolated from the pigment-producing Streptomyces bacteria early in the 1960s. Currently, it is clinically used to treat solid tumors of the breast, bile ducts, prostate, uterus, ovary, stomach, and liver, as well as hematological malignancies such as lymphoblastic leukemia [9].

Integrating 3D cell culture systems with nanotechnology-based drug delivery systems can contribute to bridging the gap between in vitro models and in vivo conditions that ultimately advance the translational potential of cancer therapies. Therefore, this study aims to investigate the performance of liposomal drug delivery systems in both 2D and 3D cell culture models. Additionally, the study seeks to validate the 3D model as a physiologically relevant platform that better replicates the tumor microenvironment compared to traditional 2D assays, offering a more accurate tool for preclinical drug testing.

## 2. Materials and methods

### 2.1. Cell lines and reagents

Human glioblastoma cell line (U-87MG) (ATCC HTB-14) and Human dermal fibroblast cell line (HDF) (ATCC PCS-201-012) were obtained from ATCC. Collagen type I and Doxorubicin were purchased from Sigma-Aldrich (Poole, UK). Doxil^®^ 20mg\10ml was purchased from Johnson & Johnson (USA).

### 2.2. Cell culture condition

U-87-MG and HDF were cultured in Dulbecco’s Modified Eagle Medium (DMEM) supplemented with 100 mg/mL streptomycin, 100 U/mL penicillin, 2 mM L-glutamine, and 10% fetal bovine serum. The cell lines were incubated at standard culture conditions at 37°C in a humidified atmosphere containing 5% CO₂. Cells were subcultured at a 70% confluency.

### 2.3. Spheroid formation

Tumor spheroids were generated using the hanging drops technique by seeding U87-MG and HDF cells at a ratio of 70:30 (1 × 10^4^ cells/drop) [10–12]. The drops were loaded into the lid of Petri dishes and incubated at 37 °C, 5% CO_2_ for 3 days. To maintain humidity, 5 mL of Phosphate Buffered Saline (PBS) was added to the bottom of the dish. After incubation, the formation of spheroids was monitored and prepared for loading into agarose for further cellular experiments.

### 2.4. 3D cellular uptake studies

#### 2.4.1. Cellular uptake of 3D spheroids by flow cytometry.

Agarose gel (0.125%) was prepared by weighing an adequate amount of agarose powder and dissolving it in an appropriate volume of cell culture medium. Wells of a 24-well culture plate was loaded with 250 µL of agarose solution as a bottom layer. After agarose solidification, spheroids from hanging drops were transferred into each well. Another 250 µL of agarose solution was added to each well and set to solidify. Finally, 500 µL of medium was added to each well. Spheroids were treated with free Doxorubicin (FD) (1 µM and 2 µM) and Doxil^®^ (1 µM and 2 µM Doxorubicin equivalent) and incubated for 4 h and 24 h. After incubation, 13 Spheroids were extracted from each well. Spheroids were disaggregated using 0.5% trypsin. After trypsinization, cells were centrifuged at 300xg for 5 min, and the pellet was resuspended in 200 µL PBS. Then, 1 × 10^4^ events were counted using FACS Canto II and analyzed with BD FACSDiva™ software version 8.0 (BD, USA). The excitation\emission wavelengths (λ 470/λ 595 nm) for Doxorubicin were used to measure fluorescence intensity (FI). The previous experiment was performed in triplicates.

#### 2.4.2. Cellular uptake of 3D spheroids by confocal microscopy.

Spheroids were incubated and treated in an agarose gel medium, as previously mentioned in Section 2.4.1. The spheroid was then transferred into a shape-untreated well plate. 4% formalin was added to each spheroid for fixation. (1:1500) DAPI\PBS of DAPI stain was added for each well and incubated in the dark. After 10 min, spheroids were ready to be replaced on a clean glass slide. A small amount of Dako mounting medium as a drop was added and incubated for 15 min before the Z-stacking examination by Zeiss LSM780 confocal microscope system (Carl Zeiss AG, Germany).

### 2.5. 2D cellular uptake studies

#### 2.5.1. 2D cellular uptake by flow cytometry.

In each well of 12-well plate, 1.25 × 10^5^ U87-MG cells were seeded and incubated for 24h. 1mL of (1 µM and 1 µM) FD and Doxil^®^ was added and incubated for 4 and 24h. All the previous experiments were performed in a triplicate. 0.5% trypsin was used to harvest and collect the cells. The excitation\emission wavelength of Dox was detected using flow cytometry to measure FI as described in Section 2.4.1.

#### 2.5.2. 2D cellular uptake by confocal microscopy.

In this experiment, U87-MG (1.2 × 10^5^) cells were seeded into individual wells of 6-well plates containing sterile coverslips and incubated for 24 h. Next, 1 mL of FD and Doxil^®^, at concentrations of 1 µM and 2 µM, was added to each well and incubated for 4 and 24 h. This procedure was performed in triplicate. To fix U87-MG cells, 4% formalin was added, and then each well was treated with DAPI stain (diluted 1:1500 in PBS) and incubated for 15 min at room temperature in the dark. After fixation, the coverslips were placed face down on clean glass slides with a drop of Dako mounting medium. Finally, images were captured using a Zeiss Plan Apo 63x/1.40 oil lens with 405 nm and 595 nm lasers to excite the DAPI-stained nuclei and Dox. Fluorescent emission was detected at 495 nm for DAPI and 470 nm for Dox, respectively.

### 2.6. MTT assay

A 96-well culture plate was used to seed (8 × 10^3) U87-MG cells, which were incubated for 24 h. The cells were then subjected to the following treatments: untreated, FD at concentrations ranging from 62.5 to 2000 nM, and Doxil^®^ at concentrations ranging from 62.5 to 2000 nM. After 72h incubation, the treatment was removed, followed by adding 100μL of culture medium and 15 μL of 3-(4,5-dimethyl-2-thiazolyl)-2,5-diphenyltetrazolium bromide (MTT) solution. After 3h of incubation, the medium was removed, and the cells were mixed with 50μL of dimethyl sulphoxide (DMSO) to dissolve the formazan. The absorbance was measured at a wavelength of 570 nm using a microplate reader (Synergy™ HTX by BioTek Instruments Inc, USA), and the IC_50_ values were subsequently determined. All experiments were performed in triplicate.

### 2.7. 3D invasion assay

Collagen medium was prepared using a type I collagen solution derived from bovine skin [10,11,13]. The chambers of an 8-chamber cover glass were loaded with collagen medium to generate the first layer (200 µL) and allowed to polymerize. After polymerization, spheroids were placed into each chamber. An additional layer of collagen medium (200 µL) was added to each well as the second layer and allowed to polymerize. Finally, media and drugs were added as follows: untreated spheroids (control), spheroids with free Dox (FD) at concentrations of 62.5 nM and 125 nM, and spheroids with Doxil^®^ at concentrations of 250 nM, 400 nM, and 2 µM. All experiments were performed in triplicate, incubated for 4 days, and images were captured daily. Data were analyzed using ImageJ software. Spheroids were observed using an inverted light microscopy at a 10 × objective lens. Images were captured daily using Nikon Eclipse camera (TE2000-4, Panasonic Lumix DMC-GF6).

### 2.8. Wound healing assay

In a 6-well culture plate, U87-MG (250 × 10^3^) cells were seeded into each well and incubated for 24 h. After incubation, a wound (scratch) was inflicted using a 200 μL pipette tip on the cell monolayer, followed by a washing step with PBS to remove cell debris. Next, the media and drugs were replaced to treat the cells as untreated cells (control), cells with FD at concentrations of 62.5nM and 125 nM, and cells with Doxil^®^ at concentrations of 250 nM and 400nM. The cells were then incubated for 3 days, with all treatments performed in triplicate. Images of the wounds were taken at 0, 3, 6, 9, and 24h. Wounds were observed using inverted light microscopy at a 10 × objective lens. Images were captured daily using a Nikon Eclipse camera (TE2000-4, Panasonic Lumix DMC-GF6), and the wound areas were analyzed using ImageJ software.

### 2.9. Statistical analysis

A two-way ANOVA test was used to analyze the data results; the experiment was performed in triplicates (n = 3). The P values significance were determined as follows: *  P ≤  0.05, ** P ≤  0. 01, *** P ≤  0.001 and **** P ≤  0.0001.

## 3. Results and discussion

### 3.1. Spheroids formation

Tumor spheroids were formed by hanging drop technique after three days of incubation; each drop in the petri dish contained the same uniform size of spheroids. Cells of 2D culture models lack cell-cell and cell-matrix interactions, which are presented in native tumors. In contrast, 3D culture models have an opportunity to culture cancer cells alone or with different cell types. Thus, it can closely mimic the native environment of tumors [14]. U87-MG cells were co-cultured with HDF and successfully formed a compacted spheroid structure to investigate tumor behavior and interaction *in vitro*. Several studies focused on the role of tumor microenvironment, which consists of cellular and non-cellular components, and how it affects cells’ behaviors such as proliferation, migration, and therapeutic resistance [15,16]. Some of these studies illustrated the effect of co-culturing cells; breast cancer cells exhibit features reflective of ductal carcinoma when co-culturing with luminal cells, myoepithelial cells, and stromal fibroblasts as 3D model [17]. Ewing tumor closely resemble patient tumors in cell-cell junctions and proliferative index [18]. These studies’ findings show that 3D culture models allow the evaluation of tumor microenvironment effects on in vitro oncology studies.

### 3.2. Cellular uptake

#### 3.2.1. 2D and 3D cellular uptake by flow cytometry.

Flow cytometry analysis method was employed to quantify the cellular uptake and penetration of Dox in both two-dimensional (2D) and three-dimensional (3D) cell culture systems. Dox, a potent anticancer therapy, exhibits intrinsic fluorescence with an emission signal at 595nm when excited with a 470nm laser. This valuable fluorescence characteristic is an important tool for understanding the cellular dynamics and mechanisms of action in biomedical research and imaging [19–21].

**Fig 1:**
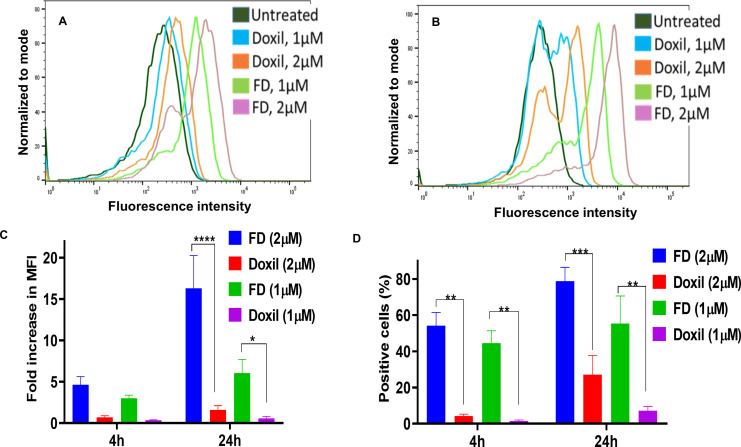
Histograms overlay and cellular uptake for 4,24h for 2D cells by flow cytometry. (A) Histogram overlay illustrating the 2D cellular uptake for 4h. (B) Histogram overlay illustrating the 2D cellular uptake for 24h. X-axis represents fluorescence intensity, while the Y-axis represents cell count. (C) Fold increase in fluorescence intensity for both con (1 µM, 2 µM) over time. *  P <  0.05, **** P < 0.0001 (two-way ANOVA). (D) Positive cells percentage for both con (1 µM, 2 µM) over time. **P <  0.01*** P <  0.001 (two-way ANOVA). Error bars indicate SD (n = 3).

**Fig 2:**
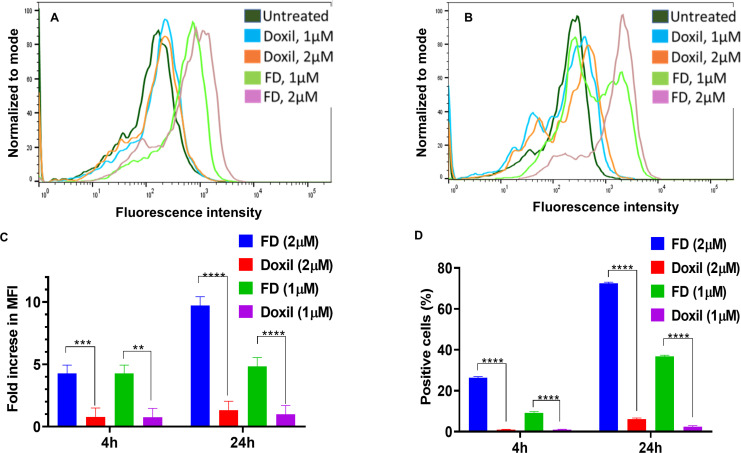
Histograms overlay and cellular uptake for 4,24h for 3D spheroids by flow cytometry. (A) Histogram overlay illustrating the 3D cellular uptake for 4h. (B) Histogram overlay illustrating the 3D cellular uptake for 24h. Fluorescence intensity is represented by the X-axis, while the Y-axis represents cell count. (C) Fold increase in fluorescence intensity for both con (1 µM, 2 µM) over time. *  P <  0.05, **** P < 0.0001 (two-way ANOVA). (D) Positive cells percentage for both con (1 µM, 2 µM) over time. **P <  0.01*** P <  0.001 (two-way ANOVA). Error bars indicate SD (n = 3).

**Fig 3:**
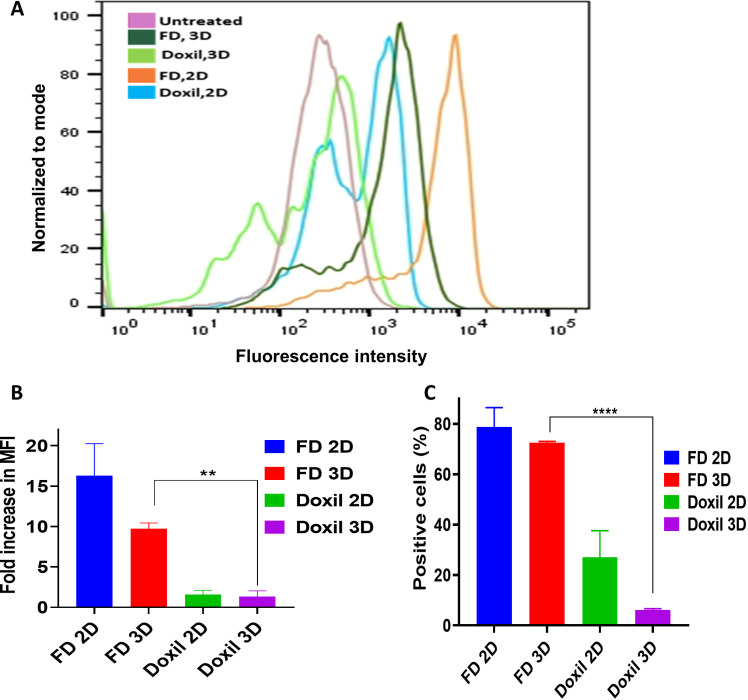
2D and 3D cellular uptake for 24h. (A) Histogram overlay for 3D cells by flow cytometry. The X-axis represents fluorescent intensity, while the Y-axis represents cell count. (B) Fold increase in fluorescence intensity for 2 µM con over time. ** P <  0.01, *** P <  0.001, **** P <  0.0001 (two-way ANOVA). (C) Positive cells percentage for 2 µM con over time. **** P < 0.0001 (two-way ANOVA). Error bars indicate SD (n = 3).

In the present study, the cellular uptake of Dox and the percentage of positive cells with Dox following the treatment with free Dox (FD) and Doxil^®^ were evaluated and compared to untreated cells control. Cells in both 2D and 3D culture systems were treated for 4h and 24h with Dox concentrations of 1 µM and 2 µM, which were selected based on clinical relevance and optimal signal detection [22,23]. Notably, the highest cellular uptake of Dox was observed at a concentration of 2 µM in both 2D and 3D models Figs 1 and 2. Moreover, the results showed that 2D cultures Fig 1 demonstrated a significantly higher Dox uptake and percent of positive cells compared to 3D cells Fig 2. Fig 3 illustrates 2D and 3D model cell uptake for 2 µM concentrations at 24 h incubation. The fold increase in fluorescent intensity was calculated as: (FIs of FD or Doxil^®^ – FI of untreated cells)/FI of untreated cells).

#### 3.2.2. 2D cellular uptake by confocal microscopy.

**Fig 4:**
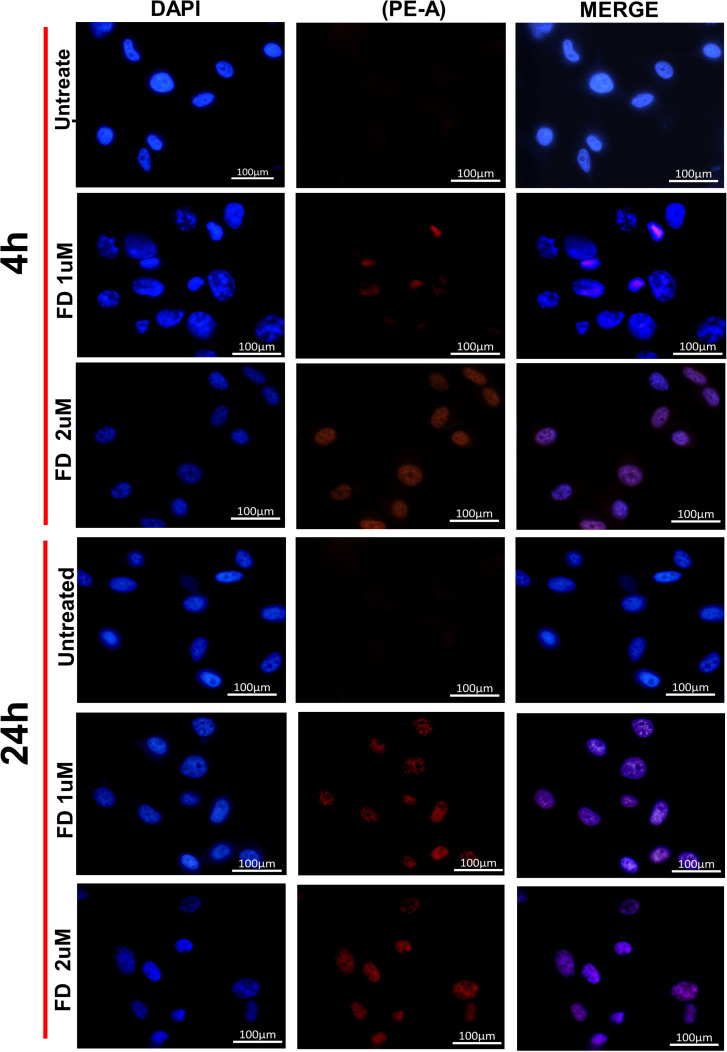
U87MG cells after 4 and 24h cellular interaction and uptake of FD by Confocal Microscopy. Localization of Dox in 1 µM and 2 µM FD treated cells. Blue fluorescence is for DAPI nuclear stain, and red fluorescence is for Dox localization. Untreated cells were used as a negative control.

**Fig 5:**
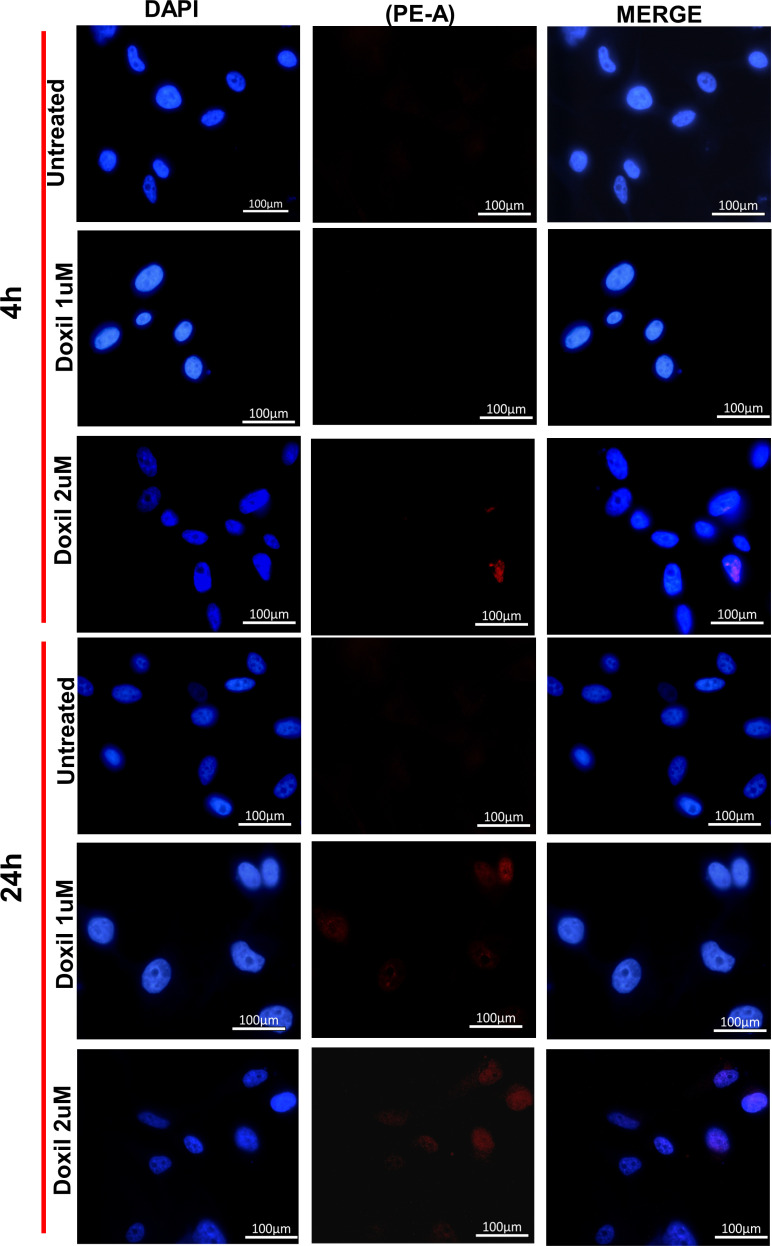
U87MG cells after 4 and 24h cellular interaction and uptake of Doxil^®^ by Confocal Microscopy. Localization of Dox in 1 µM and 2 µM FD treated cells. Blue fluorescence is for DAPI nuclear stain, and red fluorescence is for Dox localization. Untreated cells were used as a negative control.

Fluorescent microscopy visualized the localization of DAPI (blue-stained nuclei) and Dox PE-A (red signal) after cellular uptake. FD-treated cells showed more signal localization than those for Doxil^®^-treated cells, even at low concentrations, as shown in Figs 4 and 5 .

#### 3.2.3. 3D cellular uptake by confocal microscopy.

**Fig 6:**
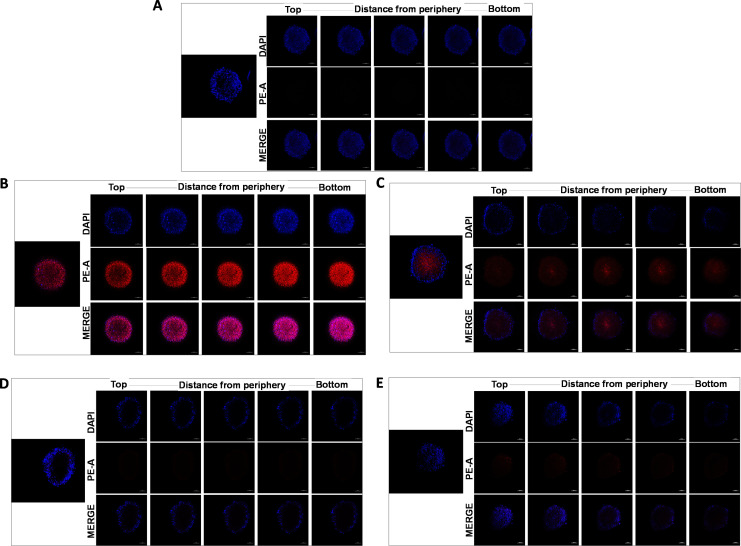
Z-stacking of U87-MG spheroids after 24h in (A) Untreated cell, (B) 2 µM FD treated spheroid (C) 1 µM FD treated spheroid, (D) 2 µM Doxil^®^ treated spheroid (E) 1 µM Doxil^®^ treated spheroid. Blue fluorescence is for DAPI nuclear stain, and red fluorescence is for Dox localization.

Spheroid’s structure could affect FD and Doxil^®^ penetration and uptake; Z-stacking by SLCM was performed to examine these interactions. Spheroids were treated with (1 µM and 2 µM) of drug concentration for 24h. FD highly diffused through the spheroid’s layer; meanwhile, Doxil^®^ localization clearly appeared within the spheroid’s surface layers. Doxil^®^ and FD interaction and diffusion are demonstrated in Fig 6.

Doxorubicin-loaded PEGylated liposome (Doxil^®^) has been broadly used to treat many cancer types [24]. Flow cytometry was applied to investigate the Doxorubicin uptake by measuring FI by cells of 2D and 3D models treated with FD and Doxil^®^. 2D and 3D cells showed a higher uptake for FD compared to Doxil^®^ in an effective manner. Several studies on the efficacy of Doxil^®^ have suggested that the mechanism behind the slow release of Doxorubicin from the liposome is not yet fully understood. The insufficient release of Dox from Doxil^®^ can be attributed to ammonium sulfate gradient loading of Doxil^®^, which results in Dox precipitation and stacking in liposomal core as bundles of fibers, and to the high cholesterol level that makes such a phase transition missed. These factors contribute to the slow Dox release, which can hinder drug passage. Thus, a small amount of drug molecules can pass the lipid membrane [25,26]. Efficacious therapy is not achieved only by targeted delivery of nanoparticles. Sufficient release of the encapsulated drug into the target tissue is equally important [3]. The flow cytometry results also demonstrated that 2D cells had a higher Doxil^®^ uptake than 3D cells; one explanation could be due to the inability of 2D monolayer cancer cells to capture the intertumoral transport (a critical mechanism of drug-tumor interaction at the tumor site) that can adequately determine the drug efficacy compared to 3D cultures [27]. To track the intracellular localization of Doxorubicin after it was taken up by the cells, its natural fluorescent property was utilized, and FM and CLSM were conducted for this purpose. For 2D monolayer cells, more Dox was detected in cells exposed to FD than those treated with Doxil^®^. This was cleared by visualizing that the red fluorescent material significantly accumulated in the nuclei of FD-treated cells, while Doxil^®^-treated cells showed less Dox accumulation. The intracellular Dox distribution seemingly depends on how the Dox was delivered to the cells. One of the possible explanations is that, after cells were exposed to FD and Doxil^®^, FD diffused more easily into cells, and because of its high affinity toward DNA, it can accumulate in the nuclei. On the other hand, many vesicles could also be transported from inside the cell to the outside by exocytosis, reducing the amount of Doxil^®^ that was taken up by endocytosis [28]. Z-stacking of 3D spheroids by CSLM was performed to evaluate the treatment’s ability to penetrate and diffuse through the spheroid structure; FD showed a broad and deep diffusion within the spheroid’s layers from the surface, whereas Doxil^®^ could not diffuse and spread deep enough as the Dox red fluorescence was visualized only on the outer surface layers (Fig 6). This can be due to the spheroid’s structure, which mimics *in vivo* tumor mass architecture, as well as NPs penetration properties since the cell’s dense packing makes a transport barrier, preventing the nanoparticle from deep diffusion into the spheroidal core; thus, NPs penetration is limited to the outer layers. This proves that spheroid represents a suitable model to test nanoparticle efficacy compared to 2D cultures [28].

#### 3.2.4. MTT assay.

**Fig 7:**
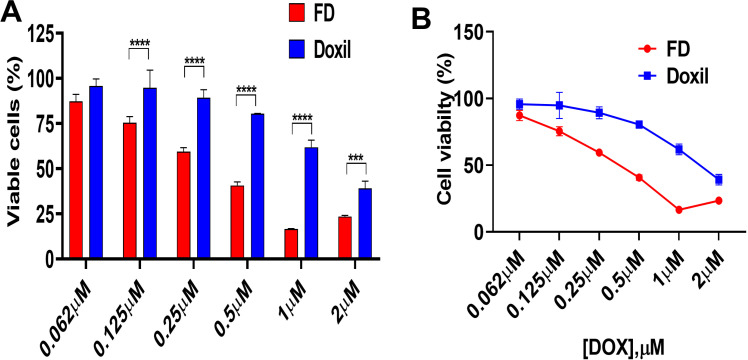
In vitro metabolic activity. (A) Comparison between FD and Doxil® treatment on U87-MG cells after 72h of incubation, as measured by MTT assay. (B) Dose- response curve of U87MG cells in FD and Doxil®. *** P <  0.001, **** P <  0.0001 (two-way ANOVA). Error bars indicate SD (n = 3).

The percentage of viable cells was evaluated after the performance of the MTT assay to estimate the treatment’s effect on cellular metabolism. The results were then calculated with respect to untreated cells (untreated, 100%) as follows: Percentage of viable cells =  (OD of treated cells/ OD of untreated cells × 100%). It is important to mention that the reduction in the MTT signal following DOX treatment may reveal DOX-mediated mitochondrial disruption, including reduced respiration and energy production, in addition to the potential effect on cell viability [29,30]. FD tended to exhibit more metabolic disruption with an IC_50_ value of 0.25 µM, while Doxil^®^ had IC_50_ of approximately 0.7 µM. Fig 7 demonstrates the cell metabolic activity for each treatment.

Doxorubicin, an antibiotic and anticancer chemotherapy agent, gains its cytotoxicity effect through the intercalation ability within DNA, inhibiting the synthesis of DNA and RNA strands. Furthermore, it inhibits topoisomerase II enzyme activity and induces apoptosis [31]. After flow cytometry and fluorescent microscopy assessment for the cellular uptake, an MTT assay was performed to evaluate the metabolic activity as an indicator of the treatment effect. Cells were treated with FD and Doxil^®^ at concentrations ranging from 2 µM con to 0.62 µM. After 72h of incubation, significant differences were observed between the treatments at different concentrations. FD showed higher metabolic disruption with an IC_50_ of approximately 250nM, while the IC_50_ of Doxil^®^ was 700nM, which could be accounted for the optimal binding and diffusivity of FD among the partial drug release and diffusion of Doxil^®^ through tissue [27].

#### 3.2.5. 3D invasion model.

**Fig 8:**
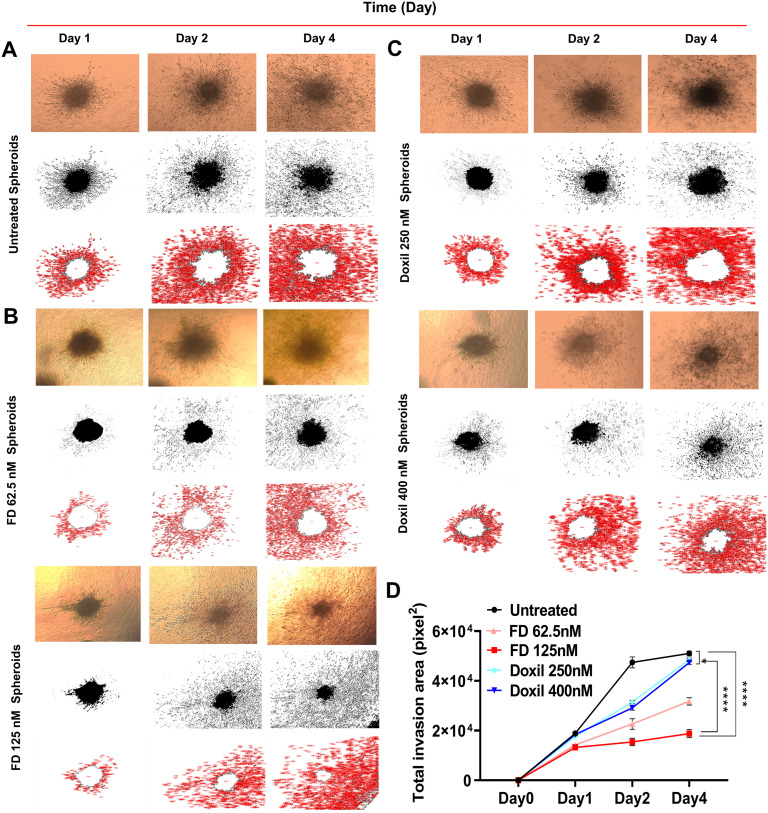
FD and Doxil^®^ treated spheroids in 3D collagen model. (A) The invasion area (represented by red area) after (62.5nM and 125nM) FD treatment for 4 days. (B) The invasion area after (250nmM and 400nM) Doxil® treatment for 4 days. (C) Comparison of FD and Doxil® invasion areas. (D)The diagram shows the relationship between the invasion area and time. The X-axis represents TIA, and the Y-axis represents times (days). *  P <  0.05, **** P <  0.0001 (two-way ANOVA). Error bars indicate SD (n = 3).

A 3D collagen invasion assay was performed to understand in vivo conditions adequately. Cells were treated with chosen concentrations lower than the IC_50_ values (125nM, 62.5nM) and (400nM and 250nM) for each FD and Doxil^®^, respectively. Also, 2 µM concentration was used for more comparable data. After that, the total invasion area (TIA) was calculated. Fig 8 shows the invasion area for FD and Doxil^®^ and compares treatments versus time. Cellular interaction with Doxil^®^ was found to be less tight than with FD. After conducting TIA measurements and analyzing the data, it was observed that the interaction between FD and cells was significantly higher than the interaction between Doxil^®^ and cells. Additionally, a minor difference was noted in the interaction between Doxil^®^-treated cells and untreated cells.

#### 3.2.6. Wound healing assay.

**Fig 9:**
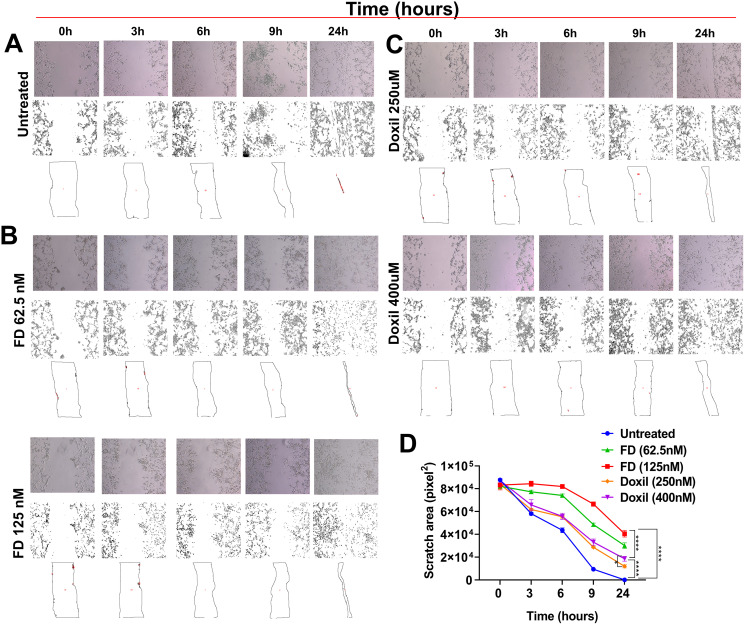
FD and Doxil^®^ treated cells in wound healing-2D model. (A) The effect of (250nM, 400nM) Doxil® concentrations on the migration area vs time. (B) The effect of (62.5nM, 125nM) FD concentrations on the migration area vs time. (C) The relation between migration area and time, and (D) the diagram demonstrates the TMA vs time comparison for FD and Doxil®. **** P <  0.0001 *  P < 0.05 (Two-way ANOVA). Error bars indicate SD (n = 3).

The wound healing assay was conducted as a 2D model in which cells were treated with specific concentrations of FD and Doxil^®^, which were below the IC_50_ values: (125nM, 62.5nM) and (400nM and 250nM), respectively. The scratch area was then calculated. Fig 9 shows that FD effectively inhibited cell migration, while Doxil^®^ was less efficient. The Figure also illustrates the correlation between the migration area following treatment with FD and Doxil^®^ and the elapsed time.

Developing a cellular model that can recapitulate tumors in vivo conditions would facilitate monitoring cell-cell communication and cell-drug interaction for a selected treatment. 3D cell culture models could offer this instead of traditional 2D ones [32]. The matrix-embedded model was chosen to represent the 3D model. U87-MG cells were cultured into 3D spheroids and embedded within the collagen matrix to mimic the ECM surrounding the tumor, followed by FD and Doxil^®^ treatment. According to the TIA observed after 4 days of incubation, there is a higher significant difference in FD-cell interaction compared to Doxil^®^-cell interaction, while less was observed between Doxil^®^-treated cells and untreated cells. Meanwhile, in wound healing assay, a chosen 2D model was performed to represent cell migration by creating a cell-free area in the monolayer cells [33]. Doxil^®^ showed the opposite of its interaction with the 3D model with the observation of a slightly higher significance in the scratch area when Doxil^®^-treated cells were compared to untreated cells. These findings could be explained according to a major aspect: the ECM consists of interconnected networks of collagen fibers that somehow could determine the diffusion complexity and block the larger nanoparticles penetration into tumors [34]. Many previous studies have proved the role of ECM on drug efficacy. One of these studies found that collagen digestion increased the drug diffusion deeper into the tumor, thus facilitating molecule delivery [35]. This finding can justify the highly significant differences between 2D Doxil® treated cells, which lack the connectivity of ECM found in the 3D model and work as a barrier for Doxil^®^ particles to diffuse and reach the spheroids [36].

## 4. Conclusion

Our study involved the use of 2D and 3D cell culture models to evaluate the efficiency of nanoparticle-based drug delivery systems. We developed a 3D model replicating the *in vivo* conditions of tumor structure and extracellular matrix to assess the delivery of liposomal nanoparticles to spheroids through a collagen matrix. This 3D model proved to be more informative than traditional 2D models as it provided a better understanding of nanoparticle interactions. We compared the interactions of liposomal-Doxil^®^ with cellular targets in both 2D and 3D models and found that the interaction of nanoparticles with 2D cells was more straightforward than that with 3D models. However, the 3D model could better recapitulate the *in vivo* microenvironment than the 2D model. Our study also revealed the need for modifications to the liposomal-Doxil^®^ to achieve sufficient drug release. We recommend additional modifications, such as surface modification for active targeting and modification in loading and encapsulation of Dox, to achieve efficient release by the delivery system. Finally, we emphasize the importance of using 3D models to understand better the interplay between nano-technological, physiochemical, and biological principles of the tumor microenvironment, which may improve the *in vivo* efficacy of nanoparticles.
